# Characterization of Courtesy Stigma Perceived by Parents of Overweight Children with Bardet-Biedl Syndrome

**DOI:** 10.1371/journal.pone.0140705

**Published:** 2015-10-16

**Authors:** Barbara Hamlington, Lauren E. Ivey, Ethan Brenna, Leslie G. Biesecker, Barbara B. Biesecker, Julie C. Sapp

**Affiliations:** 1 Rocky Mountain Cancer Centers, US Oncology, Denver, Colorado, United States of America; 2 Social and Behavioral Research Branch, National Human Genome Research Institute, Bethesda, Maryland, United States of America; 3 Metabolic Genetics and Molecular Genomics Branch, National Human Genome Research Institute, Bethesda, Maryland, United States of America; National Eye Institute, UNITED STATES

## Abstract

**Background:**

A child’s obesity is generally perceived by the public to be under the control of the child’s parents. While the health consequences of childhood obesity are well understood, less is known about psychological and social effects of having an obese child on parents. We set out to characterize stigma and courtesy stigma experiences surrounding obesity among children with Bardet-Biedl syndrome (BBS), a multisystem genetic disorder, and their parents.

**Methods:**

Twenty-eight parents of children with BBS participated in semi-structured interviews informed by social stigmatization theory, which describes courtesy stigma as parental perception of stigmatization by association with a stigmatized child. Parents were asked to describe such experiences.

**Results:**

Parents of children with BBS reported the child’s obesity as the most frequent target of stigmatization. They perceived health care providers as the predominant source of courtesy stigma, describing interactions that resulted in feeling devalued and judged as incompetent parents.

**Conclusions:**

Parents of children with BBS feel blamed by others for their child’s obesity and described experiences that suggest health care providers may contribute to courtesy stigma and thus impede effective communication about managing obesity. Health care providers may reinforce parental feelings of guilt and responsibility by repeating information parents may have previously heard and ignoring extremely challenging barriers to weight management, such as a genetic predisposition to obesity. Strategies to understand and incorporate parents’ perceptions and causal attributions of their children’s weight may improve communication about weight control.

## Introduction

Obesity and childhood obesity are increasingly significant health problems in the developed world; approximately one out of every three children in the United States is overweight or obese [1]. The medical consequences of obesity for children across the lifespan have been well documented [2,3], but the psychological and social ramifications of childhood obesity for parents of obese children have been less well-characterized [4,5,6,7]. The public generally perceives weight, particularly in children, to be under parents’ control, representing the belief that a child’s obesity is primarily due to the parent’s lack of encouraging exercise and monitoring the child’s diet. Obese children are perceived to have less individual responsibility for their overweight. Rather, their obesity is attributed to the food and exercise choices made by their parents [8]. Regardless of the perceived source of the problem, obese children are often victims of social stigmatization [9]. Reported psychological and social consequences include lower self-esteem and fewer social interactions [10].

Bardet-Biedl syndrome (BBS) is a genetic disorder characterized by childhood onset obesity, polydactyly, renal and genitourinary anomalies, blindness, and cognitive delays. This recessively inherited disorder has been associated with mutations in as many as 18 different genes [11,12]. Prioritization of weight management to offset obesity-related health risk is a major component of patient management [12]. The experiences of parents of children with BBS can provide insight into the experiences of other parents of obese children who share similar causal attributions about their child’s overweight or who perceive their child’s weight as largely beyond their ability to control and encounter similar social pressures surrounding managing their child’s weight.

Stigma is defined as a social mark of disgrace often based on appearance. Stigmatization, or the experience of negative social judgment or blame, is a well-recognized component of disease burden and efforts to ameliorate sources of stigma can lead to improved outcomes with regards to disease management [13]. Courtesy, or affiliate stigma is defined as stigma experienced by a person because of their close association with another person with a stigmatizing feature [14,15]. Courtesy stigma has been infrequently described in the literature. Coping with courtesy stigma can add a significant burden to parents of children with special needs [16,17]. Increased levels of courtesy stigma in parents of children with disabilities has been associated with reduced parental quality of life [18] and increased negative parenting [19]. As stigma plays a prominent role in the experience of living with obesity and parents are regarded by others as directly contributing to or primarily to blame for their child’s obesity, investigation of the impact of courtesy stigma in families of obese children is warranted.

Physicians and relatives are the most frequently reported sources of weight discrimination and stigmatization by obese adults [20]. Physicians and other primary care providers are often thought of as providing first-line interventions in helping patients reduce weight. Yet, obese adults often report negative interactions with their physicians [21] and parents of obese children report being concerned about being negatively judged or blamed by their child’s physician [6,22] when seeking advice to manage their child’s overweight.

Parents of children affected with BBS offered an opportunity to assess stigma and courtesy stigma associated with aspects of the condition, including obesity. We undertook a qualitative interview study to better characterize the experience of courtesy stigma, its sources and parents’ responses.

## Materials and Methods

### Ethics Statement

Participants were recruited from a study of the phenotype and metabolic characteristics of patients with BBS at the National Institutes of Health (NHGRI protocol 04-HG-0123). The National Human Genome Research Institute Institutional Review Board (IRB) reviewed and approved all components of the natural history and interview study and written informed consent was obtained from participants and/or the parents/legal guardians of minor children. The IRB approved an additional verbal consent procedure which was similarly obtained prior to parents’ participation in the interview study. BH and other study personnel obtained and documented this verbal consent in a secure database prior to conducting the interviews.

### Participants

English-speaking mothers and fathers of children 18 years old or younger with genetically-confirmed Bardet-Biedl syndrome (i.e., homozygotes or compound heterozygotes for two mutations in a gene known to cause BBS were eligible to participate. Although obesity is a hallmark characteristic of BBS, not all children with the disorder have an elevated BMI (obesity affects 72–92% of individuals with BBS [12]); only parents of at least one child with BBS with a BMI greater than or equal to 25 were eligible to participate. For families with more than one child with BBS, parents were asked to consider their youngest affected child as the subject of the interview.

### Study Design and Data Analysis

Participants completed a 30–45 minute semi-structured telephone interview designed to capture the diagnostic odyssey parents encountered in obtaining a diagnosis of BBS, instances in which their child had experienced direct stigmatization, courtesy stigma experiences parents faced in raising a child with BBS and coping strategies they employed to manage these challenges (interview guide can be found in S1 Text). Interviews took place following at least one clinical visit to the National Institutes of Health where a clinical diagnosis of BBS was established, and, in most cases, CLIA-validated molecular genetic test results confirming the clinical diagnosis were returned to families. All interviews were conducted by BH, recorded, and transcribed. In circumstances where both parents participated in the interview process, we asked that the couple refrain from discussing information about the study until both interviews were completed.

A semi-structured interview guide was used to capture courtesy stigma experiences. To start parents were asked about the story of their child’s BBS diagnosis and to describe the perceived severity of their child’s condition in relation to other children with BBS. The interviewer then defined direct and courtesy stigmatization for parents and asked them to relate their experiences. When parents reported a stigmatizing event, they were asked to describe it and were prompted for details including what feature of their child’s BBS precipitated the stigmatization, who did the stigmatizing, and how they or their child reacted. A stigmatizing experience or an experience where a parent perceived courtesy stigma was coded only if the parent provided these specific details. They often elaborated on ways they felt better prepared to respond to such events going forward.

Transcripts of the first few interviews were analyzed to identify broad themes in participants’ responses and to further refine the interview guide. A codebook was developed based on the interview guide and revised using an iterative process. Each transcript was coded in NVivo^TM^ by LEI and EB (Kappa score of 0.88).

## Results

Twenty-nine families were asked to consider participation and at least one parent from 20 families with a child with BBS agreed to take part in the interview portion of the protocol; 28 total interviews of eight fathers and twenty mothers were completed (familial response rate – 69%). A majority of the parents were married, with an average age of 43 years. The average age of the child with BBS parents discussed in the interview was 12 years, 24 total living children and one deceased child with BBS were represented in this cohort. Participants reported experiences of stigmatization of their children and the majority encountered courtesy stigma based on features of their child’s condition. Physicians and strangers were the most common sources of courtesy stigma. Parents used both direct and indirect coping strategies to manage the emotions generated by their experiences of courtesy stigma. Initials following quotes indicate parent (M for mother and F for father) and child sex (B for boy and G for girl) and age in years.

### Social stigmatization of children with BBS

All but two parents interviewed mentioned at least an emotional response to perceived differential treatment of their child, even if they did not supply specific details about the feature of BBS that provoked stigmatization and the source. Twenty parents reported specific instances of social stigmatization of their children. Parents reported that their child’s overweight was the feature of BBS that provoked direct stigmatization most often, but children’s vision problems, learning difficulties, and behavioral differences also elicited stigmatization.

*“It’s more kind of her weight-related issues and the bullying and the name-calling that kind of goes along with being different and in particular her weight…It has not necessarily been that she goes–leaves the classroom and goes–to the special classes for her special needs as far as*, *you know*, *her eyesight and stuff*. *They seem not to key in on that so much*, *but just mainly bullying and whatnot in regards to her weight*, *bullying and name calling for her weight*.*” (F/G12)*

*“At school and with other children approximately his age*, *his immaturity*, *his whining*, *and his temper tantrums have created a situation where other kids of his age now don’t really want to play with him*.*” (M/B14)*

Sources of direct stigmatization of children with BBS reported by their parents include children’s friends and classmates, strangers, family members, and healthcare professionals such as physicians and therapists.

”*A student had a birthday party and was giving out invitations to the whole classroom*. *They gave everyone one except for my son…my son asked the little boy*, *‘Where is my invitation*?*’ and the little boy said*, *‘I don’t want any stupid kids coming to my party*,*’ and he came home crying*, *things like that*. *And when the kids bring snack food*, *they won’t give him any*.*” (M/B11)*

### Courtesy stigma encountered by parents

A majority (n = 18) of parents described at least one instance of differential treatment and/or feeling negatively judged by others based on their child’s BBS feature(s). A child’s obesity was the characteristic that most frequently prompted a perception of courtesy stigma as reported by 18 parents, although a few parents described courtesy stigma based on their child’s behavior (n = 6), learning difficulties (n = 1), poor vision (n = 3) or other special needs (n = 1).

Parents’ experiences with courtesy stigma included a variety of examples of differential treatment by others including: intrusive inquiries, devaluing remarks, staring, and pointing. Most intrusive inquiries addressed their child’s overweight while devaluing remarks addressed a broader range of attributes including behavior and management of vision loss. Weight, the use of adaptive equipment such as canes, and the child’s behaviors all provoked experiences with staring and pointing.

*”I have been looked at as a parent who maybe can’t control their children because with their vision they can’t see where they are going sometimes and they will knock into somebody or*, *you know*, *they will trip over something or they will knock against something in a store and*, *you know*, *you get these looks as in*, *you know*, *‘Gosh*, *you know*, *you don’t teach your children where to go*.*’” (M/G16)*

*“There’s the behavior when you are out at the store*, *the crying*. *People will look at you like*, *you know*, *“Get your kid under control*, *lady*,*” and you can’t*. *They have that emotional immaturity and*, *you know*, *[they] cry very easily and he talks very loud and his voice intonation is not what it should be and*, *you know*, *people look at you then*.*” (M/B11)*

Parents commonly described perceptions of being “judged” as a “bad parent” by others and strongly sensed that their child’s obesity was perceived by others to be the parent’s responsibility and under their control. Our participants found negative evaluations of their parenting to be particularly distressing because of the genetic factors that contributed to their child’s obesity.

*“I feel like people are like ‘why don’t they get that girl on a diet’ and I am just scared that she is going to go through all of that and as a parent*, *I am constantly worried about people judging us that don’t know*, *you know*, *that my daughter does have this syndrome” (F/G7)*.

Parents most commonly cited health care professionals (n = 17) and strangers (n = 14) as sources of courtesy stigma. Parents reported devaluing remarks as the most common type of courtesy stigma leveraged by health care professionals.

*“I think we get frustrated because we go to specialists and doctors and they were like*, *well you have to cut these foods out and I always feel like it’s a blame game where I feel that they either don’t believe me or that we aren’t trying” (M/B10)*.

### Interactions with healthcare providers

Parents described varied interactions with health care providers ranging from primary care providers such as pediatricians to specialists including ophthalmologists and endocrinologists. A primary characteristic of interactions with the healthcare system described by parents was a sense of frustration at lack of information/expertise about the diagnosis and how best to manage their child’s obesity in the context of a rare condition.

*“Pretty much every single doctor we have ever seen has recommended that we see a nutritionist*, *and you know*, *I keep saying it’s not just about visiting a nutritionist*, *this is not just about the way she is eating because trust me that the poor child feels deprived because she can’t eat so many things other kids can…her body just doesn’t handle it like everybody else does*.*” (M/G16)*

*“Well*, *I wish there was*, *you know*, *somewhere you could go and I mean even the doctors we talk to*, *I mean*, *even our primary physician–I don’t think he*, *you know*, *I think I know more about BBS than he does*.*” (F/G7)*

Parents also reported feeling devalued and inadequate when describing specific conversations with healthcare providers about their child’s weight. Although many reported suggestions and referrals made by their child’s doctors which seem entirely appropriate (e.g., referral to a nutritionist), parents were frustrated and saddened by what they perceived as being held responsible, and a lack of understanding about the cause of their child’s overweight including the limited amount of control they have over the situation.

*“It is frustrating*, *I mean I was actually in tears in one doctor’s office because he’s trying to send me to the nutritionist again” (M/G16)*

### Parental responses and coping strategies

Parents described a range of responses to the social stigma and courtesy stigma experiences, including anger/frustration, sadness, and even ceasing relationships with family members who stigmatized them or their children.

*“At least three or four years of depression for my husband and myself*. *My husband still has a really difficult time dealing” (M/B4)*

*“We have nobody that we can really talk to about it*. *My side of the family doesn’t even see anything wrong with him*. *They don’t… he is just perfect the way he is and I guess they don’t want to deal with the situation or accept it*. *(F/B17)*

*“I am getting*, *I have gotten*, *used to people looking at him*, *but it is still is not to the point that I am not so used to it that it doesn’t hurt*. *I still hurt*, *you know*, *it hurts and it still bothers me*.*” (F/B17)*

Thirteen parents reported using methods to prevent incidences of courtesy stigma from reoccurring that can be described as problem-focused coping strategies. These included explaining their child’s condition to strangers, parents, and doctors to offset their ignorance with the hope that understanding would mitigate their tendency to pass judgment. To manage courtesy stigma experienced in a doctors’ office, one parent reported bringing unaffected children along with her child with BBS to appointments with the intention to prove competency in parenting and avoid inquiries regarding her child’s weight.

*“It makes me feel like they are judging me that they think I am a bad parent*. *And honestly*, *I did feel like that’s what people thought of me*. *I knew I wasn’t doing anything different*, *but I would honestly take my older kids*, *my good skinny kids*, *along to doctor appointments to prove that I wasn’t a bad mom*. *To prove that I had skinny kids who were really smart*, *who are already potty trained*, *so they would stop judging me*, *because that would be their advice*: *why don’t you try potty training*, *why don’t you stop feeding them so much*, *why don’t you start trying to teach them to tie their shoes*, *why don’t you do this*, *why don’t you do that*. *I almost felt like I had to bring a good kid along to prove that I do those things*. *So they think I do*.*” (M/G11)*

## Discussion and Conclusions

Participants made clear that they understood their child’s obesity to be explained by BBS and they were keenly aware that this conviction differed from the perceptions of others. They perceived that others judged them to be at fault for “causing” or “allowing” their child’s obesity and they repeatedly described feelings of anger, frustration, and helplessness associated with these perceptions. Similar feelings of blame and frustration have been reported by parents of obese children without a well-characterized genetic predisposition to obesity [23]. Obesity, for this population of children, was perceived by their parents to be something that they had limited control over, while the public seems to assume that managing a child’s weight by food choice and exercise is a primary responsibility of parenthood. The tension created by these varying perceptions created a significant source of stress and isolation for participants.

Participants reported more courtesy stigma experiences about their child’s overweight from healthcare professionals than from strangers; this finding is consistent with reports by obese adults describing stigmatizing experiences in engaging with the healthcare system [24]. While few primary care providers are familiar with rare conditions such as BBS, management of childhood obesity is becoming an increasingly common component of general pediatrics practice and many children’s hospitals have special services dedicated to pediatric weight management. There is some evidence that weight management strategies such as increasing activity and reducing consumption may help individuals with BBS maintain a healthier weight [25]. Such recommendations are consistent with pediatric standard of care. For our participants these suggestions and recommendations were perceived as distressing and judgmental because these techniques were largely ineffective for their children and because the parents did not believe that they could implement them successfully to overcome their child’s genetic predisposition to obesity.

Our participants perceived their child’s overweight as something largely beyond their control, a perception reported by other parents of obese children [23]. This attribution contributed to their views that the recommendations by their healthcare providers to manage their child’s obesity were ineffective. It is possible that these parents’ internalization of objectively appropriate recommendations (such as meeting with a nutritionist) as a negative and insensitive commentary on their parenting skills stems from their feeling helpless when it comes to managing their child’s weight. Despite their endorsement of a genetic cause, feeding ones’ child is a primary responsibility of parenthood and one that likely leaves parents still feeling blamed for their child’s weight. The reactions of health care providers, although almost certainly given with good intentions, may be perceived through a lens of inadequacy that results in feelings of being judged.

This last finding provides an interesting springboard for the investigation of the relationship between parents’ causal attributions and feelings of control over their child’s obesity and their perceptions of their interactions with the healthcare system. Our participants described a cycle of ineffective communication that most commonly comprised repeated recommendations and, at the most extreme, passive-aggressive attempts to convince health care providers of parenting competence (bringing normal-weight children to an affected child’s doctor’s visit). While it is possible that some of our participants’ feelings that their health care providers were not their allies could stem from providers’ relative lack of knowledge of a rare syndrome, one can certainly imagine that parents of children whose obesity is not due to BBS may experience similar feelings of frustration, lack of trust, and blame, and that the perceptions of stigma and courtesy stigma described by our participants may be felt by parents of children whose obesity is due to a broad range of syndromic and non-syndromic causes.

### Practice Implications

Health-related stigma plays an important role in the identification and management of both rare and common disease, and the documentation of stigmatizing experiences is a valuable initial approach to developing interventions designed to alleviate the impact of health-related stigma [13]. The degree and impact of the stigmatizing experiences reported by our participants are likely to be broadly shared by parents of children whose overweight and obesity is not a hallmark feature of a genetic syndrome.

Our findings suggest that engaging parents in a discussion of their causal attributions for a child’s obesity or overweight and assessing the degree to which parents feel that they have adequate tools and skills to exert control over this aspect of their child’s healthcare may enhance communication between pediatric healthcare providers and parents. Assessment and discussion of parents’ causal attributions for their child’s obesity by health care providers such as pediatricians and dieticians may result in parents’ feeling more allied with their providers. Parents may also benefit from targeted counseling designed to help them better cope with their experiences with stigma and courtesy stigma and future investigations designed to assess the effectiveness of such interventions are warranted.

## Supporting Information

S1 Text Interview Guide. (DOC)
