# Supplementary material for: Characterization of Courtesy Stigma Perceived by Parents of Overweight Children with Bardet-Biedl Syndrome
Source: PLoS One. 2015 Oct 16;10(10):e0140705. doi: 10.1371/journal.pone.0140705 (PMC4608820; doi:10.1371/journal.pone.0140705)
Supplement: S1 Text — (DOC) [file pone.0140705.s001.doc]

Appendix B: A Guide to Interviewing Parents of BBS Probands

Participant Name: ________________________ ID: ____________ Date: ____________

The last time we talked you agreed to participate in our interview study. To review, the goal of this interview is to help us better understand what it is like to care for a child with BBS. Particularly, we are interested in how parents feel that both they and their children have been treated differently because of their child’s diagnosis of BBS. Your participation will allow us to better understand these experiences and improve the medical care and counseling services we provide to families such as your own.

Before we begin to discuss these topics I would like to review the consent form with you and answer any questions or concerns you may have regarding this interview.

[Interviewer will review the consent form with the participant. Verbal consent will be obtained and documented by the interviewer (See Appendix D)]

Thank you for agreeing to participate in our interview study. This interview will take about 50 minutes and will be divided into three sections. In the first section I will ask you questions regarding the story of how you arrived at your child’s diagnosis of BBS. Second, we will discuss situations where you felt that your child with BBS was treated differently because of his/her diagnosis. Finally, I will ask you to describe situations where you yourself have felt judged or treated differently because you are the parent of a child with BBS.

During the consenting process you agreed to have this interview recoded. Do I have your permission to begin recording this interview? ⁭Yes ⁭No

________________________________________________________________________

Signature and printed name of individual obtaining consent Date

I am now recording this interview. Please confirm for me that I have your permission to continue recording the remainder of this interview.

Thank you, we will now begin the interview.

---------------------------------------------------------------------------------------------------------------------------------**Part 1**

---------------------------------------------------------------------------------------------------------------------------------

**Diagnostic Odyssey**

Every child has a unique story about how he/she was diagnosed with BBS. So to start this interview I would like you to please describe for me the journey you took to arrive at a diagnosis of BBS starting with when you first noticed that something was different about [child’s name].

*Prompts:*

*-Tell me about when you first found out that [child’s name] had BBS. How did you react to this diagnosis?*

*-What do you feel has been the most challenging part of raising a child with BBS?*

*-In what ways has raising a child with BBS had a positive influence on your life?*

**Severity**

You have just described for me some of the positive experiences and challenges of raising a child with BBS. I would like to better understand how you view the overall severity of [child’s name]’s BBS by asking you a few questions related to this topic.

1. If you were to imagine a BBS severity scale from 1-7 with 1 meaning unaffected and 7 meaning severely affected where would you place [child’s name] on that scale?

2. If you were to again imagine a scale from 1-7 but this time 1 represents unaffected [child’s age] year old children and 7 represents severely affected [child’s age] year old children how would you rate the severity of [child’s name] BBS compared to other [child’s age] year old children with BBS?

**Self-Efficacy and Perceived Control**

Some parents of children with BBS tell us that they feel they have control over their child’s BBS symptoms [such as weight, behavior issues, or vision difficulties]. In what ways do you feel that you have control of your child’s BBS? How would you describe your confidence in your ability to control your child’s BBS features or symptoms?

1. In what areas do you feel that you are less confident in your ability to control your child’s BBS features or symptoms?

2. In what ways do you feel that you have control over your child’s weight?

3. How would you describe your confidence in your ability to control your child’s weight?

4. In what ways has your confidence in your ability to control your child’s BBS features or symptoms changed over time?

---------------------------------------------------------------------------------------------------------------------------------**Part 2**

---------------------------------------------------------------------------------------------------------------------------------

*Transition: I would now like to shift to the second part of this interview in order to discuss events where you believe that [child’s name] may have felt stigmatized because of his/her BBS.*

**Stigma**

Some of the parents we talk to have described situations where they believe their child felt judged or treated differently because of his/her BBS. This is what we call stigma. In what ways do you believe your child has felt stigmatized because of his/her BBS?

*Prompts:*

*-Have you ever been concerned that this may happen?*

*-Can you tell me a little more about this situation when you believe that your child felt he/she was treated differently because of his/her BBS?*

*-Can you describe for me what the setting was like when this event occurred?*

*-Who was the individual that you believe treated your child differently? For example, was it a classmate, teacher, or another parent?*

*-Can you describe the specific feature of BBS that your child was being stigmatized for?*

*-How did your child react to the situation?*

*-Were you present when this situation occurred?*

*-How did you react to the situation?*

*-How would you describe your ability to cope or deal with a situation when your child is treated differently because of his/her BBS?*

---------------------------------------------------------------------------------------------------------------------------------**Part 3**

---------------------------------------------------------------------------------------------------------------------------------

*Transition: I would now like to shift to the final part of this interview in order to discuss events where you may have felt that you yourself were stigmatized because your child has BBS.*

**Courtesy Stigma**

-Some of the parents we talk to have told us that they themselves have felt judged or treated differently because their child has BBS. Have you ever felt that you yourself were judged or treated differently because of your child’s BBS? If yes, how so?

*Prompts:*

*-Can you tell me a little more about this situation where you felt that you yourself were treated differently because your child has BBS?*

*-Do you ever worry that this will occur in the future?*

*-Who was the individual that you felt treated you differently? For example, was it a teacher or another parent?*

*-Can you describe for me when this situation occurred?*

*-Can you describe for me the specific feature of your child’s BBS that you felt you were begin judged for as a parent?*

*-How did you react to the situation?*

*-How would you describe your ability to cope or deal with these types of situations when you feel that you are treated differently because your child has BBS?*

*-How has your reaction to these types of situations changed over the years?*

**Courtesy Stigma: Weight**

-Now I would like to focus specifically on weight. We have heard from some parents of children with BBS that they themselves have felt judged or have been treated differently because of their child’s weight. Have you ever felt that you yourself were judged because of your child’s weight?

*Prompts:*

*-Can you please tell me more about the experience you are thinking of?*

*-Who was the individual that you felt treated you differently. For example, was it a family member, another parent, or a stranger?*

*-Can you describe to me where this event occurred?*

*-How did you react to the situation?*

*-How would you describe your ability to cope or deal with the situation when you feel that you are treated differently because of your child’s obesity?*

*-Has your reaction to these types of situations changed over the years?*

**Courtesy Stigma: Health Care Professionals**

-Some of the parents we talk to have described situations where they felt that they themselves were stigmatized or judged by a member of the health care profession. Before your child was diagnosed with BBS in what ways did you feel stigmatized by health care professionals? After your child was diagnosed with BBS in what ways did you feel that you yourself were stigmatized by members of the health care profession?

*Prompts:*

*-Do you ever worry that this will happen?*

*-How did you react to the situation?*

*-How would you describe your ability to cope or deal with a situation when you feel that a health care professional has treated you differently because your child has BBS?*

**Support**

-Before we end this interview, I would like to gain an understanding of where you obtain support when you encounter situations such as those we have discussed today. Can you tell me about the people or resources you rely on for support when you feel stigmatized as the parent of a child with BBS?

*Prompts:*

*-Who do you turn to when you need to talk about situations where you felt stigmatized for being the parent of a child with BBS?*

*-Is there a person who you feel understands your situation the best? Have you talked to this person when you are trying to deal with emotions that arise as a result of feeling stigmatized?*

---------------------------------------------------------------------------------------------------------------------------------**Closing**

---------------------------------------------------------------------------------------------------------------------------------

That is all the questions I have for you today. Thank you very much for taking the time to discuss your experiences with me. Do you have any final thoughts or opinions you would like to share before ending this interview?

Again, thank you for participating in our interview. Please feel free to contact us if you have any questions or concerns that arise. Have a nice day.

Interview Field Notes

Participant Name: ________________________ ID: ____________ Date: ____________

Interviewee:

Interviewer:
